# Associations between physical activity, fitness, cognitive and academic performance in Swedish adolescents: Findings from a cross-sectional study

**DOI:** 10.1371/journal.pone.0344087

**Published:** 2026-03-09

**Authors:** Karin Kjellenberg, Rui Wang, Jonna Nilsson Horre, Björg Helgadóttir, Örjan Ekblom, Amika Singh, Gisela Nyberg

**Affiliations:** 1 Department of Physical Activity and Health, The Swedish School of Sport and Health Sciences, Stockholm, Sweden; 2 Division of Clinical Geriatrics, Department of Neurobiology, Care Sciences and Society, Solna, Sweden; 3 Wisconsin Alzheimer’s Disease Research Center, University of Wisconsin School of Medicine and Public Health, Madison, Wisconsin, United States of America; 4 Aging Research Center, Karolinska Institute and Stockholm University, Solna, Sweden; 5 Department of Clinical Neuroscience, Karolinska Institutet, Stockholm, Sweden; 6 Department of Neurobiology, Care Sciences and Society, Division of Nursing, Karolinska Insitutet, Huddinge, Sweden; 7 Mulier Instituut, Utrecht, Netherlands; 8 Department of Movement, School and Sports, Windesheim University of Applied Sciences, Zwolle, Netherlands; 9 Department of Global Public Health, Karolinska Institutet, Stockholm, Sweden; Kasetsart University Faculty of Veterinary Medicine Kamphaengsaen Campus, THAILAND

## Abstract

**Background:**

Adolescence is a crucial phase for the development of cognitive abilities linked to academic performance. Factors such as physical activity (PA) and fitness have been hypothesized to be linked to these outcomes, although the evidence is inconclusive. This study aimed to examine the associations between PA, fitness, academic, and cognitive performance in the same sample of adolescents.

**Methods:**

In this cross-sectional study, 1139 Swedish adolescents (mean age 13.4 years) participated. We assessed PA (accelerometry), cardiovascular fitness (submaximal ergometer test), cognitive performance (computer-based test assessing episodic and working memory with 3 tests per domain), and academic performance (grades in math and language, Swedish). We utilized multilevel mixed models to explore associations and structural equation modeling to perform mediation analyses.

**Results:**

We found a weak positive association between cardiovascular fitness and math grades (b: 0.18, 95% CI: 0.09, 0.26 β: 0.18), language grades (b: 0.16, 95% CI: 0.07, 0.25 β: 0.13), and cognitive performance (b: 0.01, 95% CI: 0.00, 0.02 β: 0.11). Further, cognitive performance scores mediated 40% of the associations between fitness and math, and language grades. Adolescents with parents with short education (≤12 years of education) or foreign-born had lower fitness levels and grades in math and language (all p < 0.05).

**Conclusion:**

Fitness was the only significant factor associated with cognitive and academic performance, although the association was weak. Given our findings of low fitness and academic performance among adolescents with parents with short education or parents born abroad, future studies should tailor their interventions to ensure the inclusion of these groups.

## 1. Introduction

Adolescence is a crucial stage of development where important social, emotional, and higher cognitive skills are acquired. These skills are essential for preparing individuals to succeed in different aspects of society, such as in school or work life [1].

Therefore, adolescence is considered a critical period for brain development, during which environmental and behavioral factors are known to play a significant role [1,2]. Physical activity (PA) has been suggested as a potential factor that could benefit cognitive and academic performance; nevertheless, most adolescents do not meet the PA recommendations. For instance, only 32% of Swedish adolescents (aged 11–16 years) met the recommendations [3]. When reviewing previous studies in the field, it is important to acknowledge that while cognitive and academic performance are often discussed together, they are distinct concepts [4]. Cognitive abilities are shaped by many factors, such as age [5], parental and environmental factors [6], and genetics [7]. These skills include for example working memory and episodic memory, which are essential for academic performance [5,8]. For instance, episodic memory facilitates the storage and retrieval of information within specific contexts [8], while working memory aids in organizing ideas, problem-solving, planning, and goal achievement [5]. However, academic performance is also shaped by other factors, including a student’s engagement and participation in the school environment, as well as the quality and quantity of education received [4].

Several theories propose the mechanisms by which PA improves cognitive performance, such as alterations in grey and white matter, as well as improving neural properties like functional activation or connectivity, which are crucial for cognitive performance [9] and thereby, academic performance. However, the evidence in this area is primarily based on studies conducted on animals or adults. While a small-scale PA intervention in children study found that PA participation increased white matter integrity and that these improvements were associated with improved cognitive performance [10], there are insufficient studies to establish a link in adolescents.

Habitual PA participation, more specifically vigorous-intensity PA (VPA) enhances cardiorespiratory fitness. For example, a review found that school-based PA interventions improved cardiorespiratory fitness among adolescents [11]. Consequently, fitness is proposed to be a factor in the causal pathway linking PA to academic performance. Cross-sectional studies involving children have found that fitness plays a mediating role in the association between PA and academic performance [12,13]. One suggested mechanism is that high fit adolescents allocate attentional resources more efficiently (indicated by the monitoring of the event-related potential component P3, thought to reflect this ability), resulting in better cognitive performance, compared to their less-fit counterparts [14]. Cross-sectional studies have also found fitness to have a direct positive association with cognitive [15] and academic performance [16]. Although it is important to acknowledge that cardiorespiratory fitness is influenced by pubertal status [17], highlighting the need to control for puberty status among adolescents.

While single-session PA has been shown to produce beneficial effects on some aspects of cognitive performance [2], long-term PA interventions have demonstrated low or no effect on cognitive or academic performance [15,18]. Some of the potential limitations of these interventions are low fidelity (i.e., intervention was not executed as intended), and the failure to monitor the proposed improvements in participants’ PA during the interventions [18]. This makes it challenging to discern whether null results in PA interventions arise from PA not enhancing cognitive and academic performance, or from the intervention failing to increase PA levels, thus hindering investigation into the impact of PA [18].

While the inconclusive results could be due to the failure to induce changes in PA habits, cross-sectional studies investigating the association between habitual PA and cognitive or academic performance have also shown mixed results. Although positive associations have been found in some aspects of cognitive performance, mainly executive functions, as well as academic performance [2,15,19]. Previous studies have limitations, such as lack of robust measures for PA and cognitive performance and the omission of key confounders.

A review concluded that the accelerometer-based studies showed inconclusive associations between PA and academic performance, while self-reported studies yielded more consistent results, although many of them overlooked important confounding variables [19]. Yet, relying on self-reported PA limits the possibility to distinguish between intensities and adolescents tend to over-report their time spent in PA. For instance, one study showed that only 7–20% of self-reported MVPA and VPA could be confirmed using accelerometry [20].

Another aspect is that studies that encompass both children and adolescents are frequently discussed collectively although most studies have been performed in children, with limited research focusing specifically on adolescents. This is problematic considering age has been proposed as a moderating factor [21]. For instance, intervention studies suggest larger effects in participants under 12 years old [22]. Additionally, some research suggests that individuals with lower baseline performance may experience greater benefits from physical activity interventions on cognitive or academic performance [23,24]. Furthermore, positive effects of exercise have also been observed on cognitive behaviors in individuals with Attention Deficit Hyperactivity Disorder [25].

Moreover, there is a lack of studies examining the effects of different PA intensities separately. A cross-sectional study in children, for example, found a positive association between VPA and academic performance, but no such association between MPA and academic performance [26], indicating that the relationship may differ based on the intensity of PA. Lastly, many studies on cognitive performance rely on only one or two tasks, varying in format (e.g., letters, numbers, visual cues) and response mode (e.g., computer, oral, paper, and pencil). This limited approach may not accurately capture a cognitive skill but rather proficiency in specific tests. Moreover, most previous research has focused on executive functions [2], whereas other cognitive skills, such as episodic memory are less explored.

In conclusion, there is a scarcity of studies using detailed measures of PA and cognitive performance to investigate how PA at different intensities, along with fitness, are associated with cognitive and academic performance within the same sample. In addition, few studies have included key confounding factors, such as parental education, which is known to be strongly associated with both academic and cognitive outcomes. Furthermore, there is a need for studies exploring the mediating role of fitness and cognitive performance in these associations to gain deeper insight into how these factors covary among adolescents.

**Fig 1:**
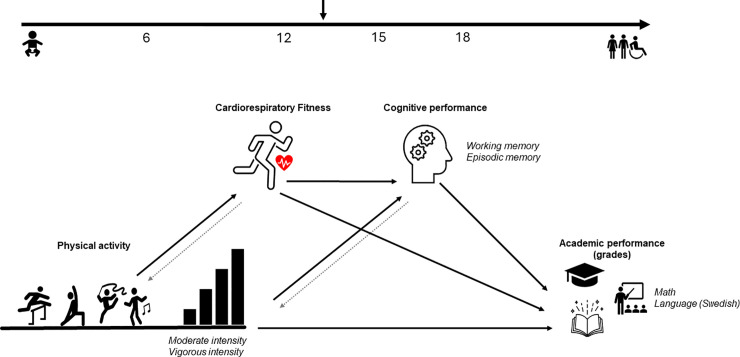
Theoretical model outlining the proposed associations between physical activity and academic performance in adolescents. The hypotheses of this study were pre-registered at: https://osf.io/ep74n. We hypothesize that physical activity of different intensities—moderate (H1) and vigorous (H2)—will be associated with cognitive performance, as well as academic performance (H3 and H4). Additionally, we propose that fitness will be associated with both cognitive (H5) and academic performance (H6). We further hypothesize that cognitive performance will serve as a mediator in the relationships between physical activity and academic performance (H7). Finally, we also hypothesize (H8) that fitness mediates the association between vigorous physical activity (VPA) and academic performance. This hypothesis was not specified in the pre-registration but was added later, based on findings from similar studies in children. The grey dotted arrows in the model represent potential reverse associations, which are not examined in this study.

The aim of this study was therefore to investigate how accelerometer-based PA (MPA and VPA) and fitness are associated with cognitive (working memory and episodic memory) and academic performance (grades in math and language) among Swedish adolescents while adjusting for gender, puberty, parental education, and parental country of birth. Additionally, we aimed to explore whether fitness and cognitive performance mediated these associations. The hypothesis and theoretical framework are shown in Fig 1.

## 2. Methods

### 2.1. Sample and study design

This study is part of the larger cross-sectional study “Physical Activity for Healthy Brain Functions in School Youth”. Approval for the study was obtained from the Swedish Ethical Review Authority (reference: 2019−03579, date of approval: 2019-08-06). All participating adolescents and their guardians provided written informed consent. In cases where written consent could not be obtained by the guardians (for instance one guardian lived elsewhere), verbal consent was obtained by phone from that guardian. This verbal consent was carefully documented and witnessed by the research staff. The design, recruitment, and protocol of the study have been described in more detail elsewhere [27].

In brief, all schools within 2–3 hours’ driving distance from the Swedish School of Sport and Health Sciences (GIH) in Stockholm, Sweden were invited to participate in the study (n = 558). The invitation was sent out by e-mail to the principals or co-principals of the schools in February 2019. A total of 84 schools accepted the invitation; however, school inclusion was capped at 40 schools, ensuring diversity in geographical location, school type, and parental education levels for practical reasons. Six schools dropped out due to time constraints, leaving a final sample of 34 schools (see supporting information S1-S3 Figs).

The schools provided a list of contact information for students in the participating classes, allowing invitation letters and consent forms to be mailed to adolescents and their guardians. Teachers collected the consent forms and informed the researchers of the number of participants from each class and school. The inclusion criteria were grade seven students, with the ability to read or understand Swedish, as the questionnaires and cognitive test instructions were in Swedish. The students were recruited between August 12^th^ – December 6^th^, 2019.

A total of 1139 grade seven students from these schools participated, representing a 73% participation rate out of 1556 eligible students. Of the 1556 eligible students, 63 were unable to participate due to illness or absence, 351 did not provide consent and therefore declined, and three were excluded because their consent forms were incomplete (e.g., missing a signature from one or both guardians).

### 2.2. Protocol

The data was collected at our research center at GIH in Stockholm, Sweden, between September 26^th^ – December 6^th^, 2019. Each day, a maximum of 60 adolescents (two groups of 30) participated.

The visit typically lasted four to five hours. Upon arrival, adolescents were given a standardized breakfast and briefed on the day’s activities. They were then split into two groups, each accompanied by at least one teacher. Participants completed a questionnaire, were equipped with a hip-worn accelerometer performed a cognitive test battery and a submaximal cycle ergometer test. The adolescents received a 300 SEK gift card (approximately 30 USD) for their participation in the study.

### 2.3. Measurements

#### 2.3.1. Cognitive performance (working memory and episodic memory).

A computer-based test battery with six tests to assess two cognitive domains: working memory (letter updating, numerical 3-back, spatial updating) and episodic memory (word recall, number-word recall and object-position recall). The test battery is described in more detail in the supporting information (S2-S6 Figs) and by [28]. The internal variability (Cronbach alpha) was 0.76 for cognitive performance (based on all tests), and 0.67 for the working memory and 0.73 for episodic memory domains.

Each test variable was transformed to T scores, centered to a mean of 50 and a SD of 10 to allow for meaningful comparisons between all outcomes. A structural equation model (SEM) was employed to generate a latent variable, incorporating loadings from all cognitive tests (available in supporting information S5 Fig). The model demonstrated a good fit, as evidenced by a Root Mean Square Error of Approximation (RMSEA) at 0.042, below the cut-off threshold of <0.06 and a Comparative Fit Index (CFI) of 0.990, exceeding the minimum criterion of 0.90. Subsequently, factor scores for each latent variable (global cognitive performance, working memory, and episodic memory) were extracted from the models and utilized as outcome measures for cognitive performance. Global cognitive performance was used as the primary outcome but findings on working memory and episodic memory are presented in supporting information S4 Table.

#### 2.3.2. Academic performance (grades in math and language).

Academic performance data was obtained by official records from Statistics Sweden, using final grades from math and language (Swedish) in grade six. The language subject has two variations: Swedish for native speakers (88% in our sample) and Swedish as a second language (12%). Within the Swedish school system, an A signifies the highest grade attainable, while an F denotes a failing grade. These variables were converted into numeric values ranging from one to six, one representing F and six representing A. The variables were transformed to T scores, centered to a mean of 50 and a SD of 10 to allow for meaningful comparisons between all outcomes (cognitive and academic performance). Grades in math and language were used as the primary outcomes, while results from a combined academic performance outcome (average grade) are presented in supporting information S5 Table.

#### 2.3.2. Physical activity (PA).

PA was assessed using a hip-worn triaxle accelerometer (model GT3X + , Actigraph, LCC, Pensacola, FL, USA) with a sampling rate of 30 Hertz. Participants were instructed to wear the accelerometer all waking hours, but not during water-based activities, for a period of seven days following the visit at the research center. The accelerometer data were processed in Actilife (v6.13.3) as uniaxial data, using 5 seconds epoch time intervals. Non-wear time was defined as 60 minutes of zero counts and no spike tolerance. An individual time filter for expected wear-time was applied to the data based on the participant’s reported awake/asleep time (extracted from the questionnaire). A minimum of 500 minutes of wear time was considered a valid day (after excluding non-wear time), and at least three valid days (at least one weekend day) were required to be included in the analyses [29]. The data were categorized using the following cut-offs counts per minute: MPA 2296–4011 and VPA ≥ 4012 [30]. Minutes spent in each intensity were divided by wear time and multiplied by 100 to create a variable representing the percentage in each intensity.

A variable for MVPA was created by combining MPA and VPA intensities. However, these results are only presented in the supporting information (S6 Table) for reference purposes, considering MVPA is commonly used in the research field but falls outside the scope of our study aim.

#### 2.3.4. Cardiovascular fitness.

Cardiovascular fitness was measured through the Ekblom-Bak test, an incremental submaximal cycle ergometer test. To estimate maximal oxygen consumption (expressed as milliliters of oxygen per kilogram of body weight per minute, mL/kg/min) the change in heart rate response between two different workloads was used while accounting for gender and age. To account for pubertal status, prepubertal boys (Tanner stage 1–2) were estimated using the equation for females, as recommended by Björkman et al, [31].

#### 2.3.5. Other variables.

Register data on parental education was provided by Statistics Sweden. The variable was dichotomized into short education (≤12 years of education, indicating completion of Swedish upper secondary school or less) or long education (>12 years of education, typically referring to university education), using the parent with the highest level of education.

In the questionnaire, adolescents provided information about their gender (girl, boy, or other) and their parental birth country (one parent born in Sweden/one parent born abroad, both parents born in Sweden, or both parents born abroad).

The adolescents’ body weight was assessed using a calibrated Tanita BC-418 scale (Tanita Corporation, Tokyo, Japan). Height was measured using a SECA 5123 stadiometer (SECA Weighing and Measuring Systems). Two weight and height measurements were taken and averaged, with the final values rounded to the nearest 0.1 kg or meters. Body Mass Index (BMI) was calculated as weight (in kilograms) divided by height squared (in meters). BMI classification was determined according to the International Obesity Task Force guidelines [32]. Pubertal status was self-reported by the adolescents using Tanner drawings [33]. Classroom noise levels during the cognitive test were rated as low, medium, or high by the research staff.

### 2.4. Statistical analysis

The statistical analysis plan and hypotheses were pre-registered at: https://osf.io/ep74n, as there were deviations from the statistical plan these have also been uploaded to the registry. Data analysis was conducted using Stata/SE version 18 (StataCorp LLC, College Station, TX, USA). Descriptive statistics were used to summarize the data, including means and standard deviations, or frequencies and proportions. Group differences (presented in Table 1, and in supporting information S2–S3 Tables) were assessed either through independent t-tests, ANOVAs, or Chi2-tests.

**Table 1:** Descriptive characteristics of the study sample by gender (*n* = 1139).

	n	missing	All	n	Girls	n	Boys	Sig.
								p
**Total** n (%)			1139 (100)		580 (51.0)		558 (49.0)	
Age (year)	1139	0	13.4 ± 0.3	580	13.4 ± 0.3	558	13.4 ± 0.4	0.147
Parental education, > 12 years n (%)	1102	37	730 (66.2)	563	371 (65.9)	538	358 (66.5)	0.821
**Parental country of birth**	1107	32		568		538		
Both parents born in Sweden n (%)			656 (59.3)		344 (60.6)		312 (58.0)	0.552
One parent born in Sweden n (%)			168 (15.2)		87 (15.3)		81 (15.1)	
Both parents born abroad n (%)			283 (25.6)		137 (24.1)		145 (27.0)	
**BMI status** ^**1**^	1134	5		580		554		0.203
Underweight n (%)			89 (7.8)		38 (6.6)		51 (9.2)	
Normal weight n (%)			815 (71.8)		430 (74.1)		384 (69.3)	
Overweight n (%)			179 (15.8)		89 (15.3)		90 (16.2)	
Obesity n (%)			52 (4.6)		23 (4.0)		29 (5.2)	
**MPA**								
MPA (average min per day/week)	904	235	30.6 (9.8)	490	29.8 (9.0)	413	31.4 (10.7)	**0.012**
% MPA (average percentage per day/week)	904	235	3.9 (1.3)	490	3.8 (1.1)	413	4.0 (1.3)	**0.004**
**VPA**								
VPA (average min per day/week)	904	235	21.4 (12.0)	490	19.7 (11.61)	413	23.40 (12.2)	**<0.001**
%VPA (average percentage per day/week)	904	235	2.7 (1.5)	490	2.5 (1.5)	413	3.0 (1.5)	**<0.001**
**Accelerometer wear time**								
Wear time (average week)	904	235	792.6 (60.8)	490	796.0 (58.1)	413	788.5 (63.8)	0.067
Total included valid days	904	235	6.0 (1.1)	490	6.2 (1.0)	413	5.8 (1.1)	**<0.001**
**Fitness** ^2^								
Estimated V0_2_ max (mL/kg/min)	1020	119	49.5 (10.1)	569	44.8 (8.5)	451	55.5 (8.9)	**<0.001**
**Working memory score**								
Letter updating (max: 48)	1135	4	35.46 (7.6)	578	36.4 (6.9)	556	34.45 (8.2)	**<0.001**
Numerical nback (max: 108)	1136	3	74.4 (26.9)	578	76.6 (24.7)	557	72.13 (28.8)	**0.005**
Spacial updating (max: 30)	1136	3	12.1 (6.5)	578	12.1 (6.0)	557	12.16 (7.04)	0.795
**Episodic memory score**								
Word recall (max: 32)	1106	33	15.8 (5.9)	568	16.9 (5.8)	537	14.7 (5.8)	**<0.001**
Number-word recall (max: 20)	1125	14	3.2 (2.8)	574	3.6 (2.8)	550	2.8 (2.6)	**<0.001**
Object-position recall (max: 24)	1115	24	13.0 (5.2)	570	14.1 (4.9)	544	11.9 (5.3)	**<0.001**
**Math grade (final grade)**	1069	70		547		521		0.467
A, n (%)			140 (13.1)		68 (12.4)		72 (13.8)	
B, n (%)			201 (18.8)		109 (19.9)		92 (17.7)	
C, n (%)			212 (19.8)		106 (19.4)		106 (20.4)	
D, n (%)			222 (19.8)		122 (22.3)		99 (19.0)	
E, n (%)			216 (20.2)		108 (19.7)		108 (20.7)	
F, n (%)			78 (7.3)		34 (6.2)		44 (8.5)	
**Language, Swedish, grade (final grade)**	1167	72		546		520		**<0.001**
A, n (%)			99 (9.3)		72 (13.2)		27 (5.2)	
B, n (%)			224 (21.0)		145 (26.6)		79 (15.2)	
C, n (%)			267 (25.0)		149 (27.3)		118 (22.7)	
D, n (%)			236 (22.1)		104 (19.1)		131 (25.2)	
E, n (%)			170 (15.9)		59 (10.8)		111 (21.4)	
F, n (%)			71 (6.7)		17 (3.1)		54 (10.4)	

The descriptives are mean ± standard deviations, unless otherwise specified.

Group differences between boys and girls were analyzed with a t-test (continuous variables) or Chi^2^ test (categorical variables).

^1^ BMI status; Body Mass Index status (defined according to IOTF 2012), ^2^Fitness; Estimated V0_2_ max expressed in mL/kg/min, Abbreviations: BMI; Body Mass Index, MPA; moderate physical activity, VPA; VPA vigorous physical activity.

Multilevel mixed effect model was used to study the associations between PA (MPA or VPA), fitness and cognitive or academic performance (grades in math or language), modeling school as level 1 and individual adolescents as level 2. A random intercept was introduced for each school to account for variations at the school level while the slopes were fixed. This approach was based on the assumption that the associations between PA or fitness and cognitive or academic performance would be consistent across schools. Overall, the intraclass correlation coefficient showed that between 8–20% of the variance in the academic performance models could be attributed to the clustering of schools. The analyses only included cases with complete data, that is, participants with no missing values for any variables included in the models. As multiple models were estimated (e.g., MPA, VPA, and fitness across several outcomes), we adjusted for multiple comparisons using the Benjamini–Hochberg False Discovery Rate (FDR) approach, applying a false-discovery rate of 5% [34], which resulted in a corrected significance threshold of alpha below 0.018. This procedure was implemented to control for multiple testing and to reduce the risk of Type I error.

Extreme outliers in all variables were identified using the “Outlier labeling rule”, which calculates upper and lower cut-offs based on the formula: upper = Q3+(3*(Q3-Q1)) and lower = Q1-(3*(Q3-Q1)) [35]. However, no extreme outliers were identified in any of the variables, therefore, no observations were excluded based on this rule. The assumptions for mixed models were checked. In certain models, the assumptions of normal distribution and homoscedasticity were violated. To address this, we employed robust estimates. All models were run both crude and adjusted (including gender, puberty status, parental education, and parental country of birth, based on previous literature). The adjusted results will serve as the basis for interpretation, while the crude results are included for reference. Given the interconnected nature of these exposures and the study’s focus on examining the association of each exposure with the dependent variable, rather than their collective influence, we chose to test each exposure in separate models.

To further examine the associations, we performed path analysis using SEM. These models explored whether cognitive performance mediated the significant associations between the exposures and academic performance. In addition to the pre-registered hypotheses, we conducted an exploratory analysis to test a pathway (H8) suggested by prior literature, that fitness mediates the association between VPA and academic performance [12,13]. Although the association between VPA and academic performance was not statistically significant, we decided to explore fitness as a potential mediator, given the link between VPA and fitness. MPA was not tested as an exposure in the mediation analyses. We tested both mediation analyses within the same model: (1) the association between VPA and academic performance, mediated by fitness, and (2) the association between fitness and academic performance, mediated by cognitive performance, as the models were assumed to be correlated and not independent (see the theoretical outline in Fig 1). The mediation models indicated good model fit (RMSEA = 0.028 and CFI = 0.999 in the math model and RMSEA = 0.026 and CFI = 0.999 in the language model). To assess the indirect and direct effects we used the Medsem package in Stata, following the Barron and Kenny approach to test mediation, using the modified significance test by Iacobucci et al. using the Monte Carlo method with 2500 samples, utilizing a significance level of p < 0.05, this approach has been described in more detail by Mehmetoglu [36].

Sensitivity analyses were performed, excluding participants with the latest start in the cognitive test battery (as this group exhibited lower scores), comparing the results to those including all participants. Furthermore, sensitivity analyses were also conducted including participants with at least one valid day of accelerometer measurement, compared with the stricter criterion of at least three valid days. Sensitivity analyses were also conducted to explore the influence of classroom noise level by comparing the results between the models with and without this confounder.

## 3. Results

### 3.1. Participants

A total of 1139 adolescents (mean age 13.4 ± 0.3) participated in the study, 51% were girls (see Table 1 for characteristics of the participants). Regarding their parents, two-thirds (66%) had parents with more than 12 years of education, 59% had both parents born in Sweden and 26% had both parents born abroad (most were born outside Europe, 68% of the mothers and 63% of the fathers).

### 3.2. Descriptive statistics for cognitive scores, academic performance, physical activity, and fitness

With regard to the cognitive test battery, 1082 adolescents completed all tests. Descriptive details of the cognitive test parameters, including start times, test duration, and classroom noise levels, are provided in the supporting information, S1 Table. The correlation between cognitive and academic performance was moderate r(1067)=0.59 for math and r(1065)=0.55 for language.

To assess the confounders, we compared mean differences in the dependent and independent variables between groups based on gender (Table 1), parental education, and parental country of birth (supporting information S2-S3 Tables). Girls had higher scores in all cognitive tasks (except in special updating) and grades in language (but not math) compared to boys (see Table 1). Further, adolescents with parents with long education, or both parents born in Swedish had higher cognitive scores and higher grades (supporting information S2-S3 Tables).

With regards to fitness and PA, 1020 adolescents had valid fitness measurements, while 904 had at least three days of valid accelerometer measurements. Those with valid accelerometer measurements had higher scores on the cognitive tasks (p < 0.05 for all tests) and math grade, there were no significant differences in language grade or fitness level, compared to those with less than three valid days.

Furthermore, boys spent more time in MPA and VPA and had higher fitness, compared to girls (Table 1). Additionally, adolescents to parents with long education spent more time in MPA (but similar time in VPA) and had higher fitness, compared to adolescents with parents with short education (≤12 years of education). There was no difference in PA depending on parental country of birth. For fitness, adolescents to parents with long education (>12 years of education) or both parents born in Sweden had higher fitness, compared to adolescents with parents with short education and both parents born abroad.

### 3.3. Associations between fitness and cognitive and academic performance

There was a significant association between fitness and cognitive performance, in the adjusted models (see Table 2). Thus, confirming our hypothesis that fitness would be significantly associated with cognitive performance (H5).

**Table 2:** Associations between exposures and cognitive performance scores analyzed with multi-level linear regression with schools as a cluster.

Cognitive performance				
Crude	n	b (95% CI)	Sig (p)	β
%MPA	864	−0.06 (−0.11, −0.01)	0.031	−0.09
%VPA	863	0.00 (−0.04, 0.05)	0.852	0.01
Fitness	980	0.00 (−0.00, 0.01)	0.860	0.01
**Adjusted** ^1^	n	b (95% CI)	Sig (p)	β
%MPA	766	−0.02 (−0.07, 0.03)	0.383	−0.03
%VPA	765	0.02 (−0.01, 0.06)	0.203	0.04
Fitness	865	**0.01 (0.00, 0.02)**	**0.008**	**0.11**

Coefficients: b= unstandardized and β= standardized; CI; confidence interval.

^1^ The adjusted model included parental education, parental country of birth, pubertal status, and gender as confounders.

Working memory and Episodic memory are based on factor scores from a SEM model loading from 2 latent factors: episodic memory and working memory, with 3 tests in each domain.

Abbreviations: Fitness; Estimated V0_2_ max expressed in mL/kg/min, %MPA; percent spent in moderate physical activity, %VPA; percent spent in VPA vigorous physical activity.

Significant results below the adjusted alpha level of 0.018 are highlighted in bold.

Furthermore, a significant association was observed between fitness and academic performance (both math and language grades) in the adjusted models (Table 3). This supports our hypothesis (H6), suggesting that adolescents with higher fitness levels also achieved higher grades.

**Table 3:** Associations between exposures and academic performance (math or language) analyzed with multi-level linear regression with schools as a cluster.

Academic performance								
	Math				Language (Swedish)			
Crude	n	b (95% CI)	Sig (p)	β	n	b (95% CI)	Sig (p)	β
%MPA	854	−0.54 (−1.00, −0.09)	0.020	−0.07	852	−0.70 (−1.29, −0.12)	0.018	−0.09
%VPA	853	0.40 (−0.15, 0.95)	0.155	0.06	851	0.20 (−0.37, 0.77)	0.493	0.03
Fitness	958	**0.13 (0.07, 0.18)**	**<0.000**	**0.13**	956	−0.01 (−0.07, 0.05)	0.804	−0.01
**Adjusted** ^1^	n	b (95% CI)	Sig (p)	β	n	b (95% CI)	Sig (p)	β
%MPA	774	−0.44 (−0.84, −0.04)	0.032	−0.06	772	−0.24 (−0.78, 0.21)	0.395	−0.03
%VPA	773	0.39 (−0.16, 0.94)	0.058	0.07	771	0.54 (0.04, 1.04)	0.036	0.08
Fitness	869	**0.18 (0.09, 0.26)**	**<0.000**	**0.18**	867	**0.16 (0.07, 0.25)**	**0.001**	**0.13**

^1^ The adjusted model included parental education, parental country of birth, pubertal status, and gender as confounders.

Coefficients: b= unstandardized and β= standardized; CI; confidence interval.

Abbreviations: Fitness; Estimated V02 max expressed in mL/kg/min, %MPA; percent spent in moderate physical activity, %VPA; percent spent in VPA vigorous physical activity.

Math and Swedish are based on the final grade in grade 6 (T-scores).

Significant results below the adjusted alpha level of 0.018 are highlighted in bold.

### 3.4. Associations between physical activity (MPA or VPA) and cognitive and academic performance

As seen in the adjusted models (Table 2 and 3), there were no significant associations between MPA and cognitive or academic performance (grades in math or language). Therefore, we rejected our hypothesis that MPA would be significantly associated with cognitive (H1) or academic performance (H3).

Further, there were no significant associations found between VPA and cognitive or academic performance in the adjusted models (Table 2 and 3). Therefore, we rejected our hypothesis, that VPA would be significantly associated with cognitive (H2) or academic performance (H4).

### 3.5. Path analyses using fitness and cognition as mediators

**Fig 2:**
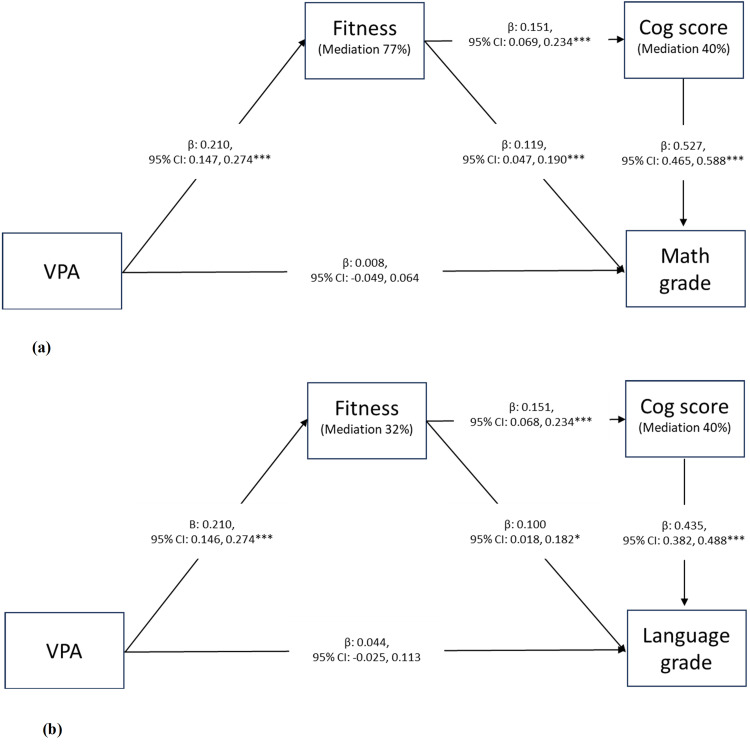
The mediating role of fitness on the association between VPA and grades in math (a) or language (b), and cognitive performance (cog score) on the association between fitness and grades in math (a) or language (b). Mediation analyses were adjusted for gender, parental education, parental country of birth, pubertal status and gender. To account for the clustering of schools the vce(cluster) option was applied in Stata. Fitness was found to mediate the association between VPA and math **(a)**, and language (b) grade. Cognitive performance was found to mediate the association between fitness and math (a) and language (b) grade. *** p < 0.001; * < 0.05, n = 688 math model, n = 687 language model.

Fitness and cognitive performance were explored as mediators using SEM, these results are presented in Fig 2a and b.

We found individual cognitive performance differences to significantly mediate the association between fitness and academic performance, supporting our hypothesis (H7). In the math models, cognitive performance accounted for 40% of the association (Fig 2a). Further, in the language model, cognitive performance accounted for 40% of the association (Fig 2b). In both models, the direct association between fitness and academic performance was still significant, after accounting for the mediators and confounders.

Furthermore, we found fitness to significantly mediate the association between VPA and academic performance (Fig 2b).This finding supports our exploratory hypothesis (H8).In the math model, fitness explained 77% of the association between VPA and math, but the direct association between VPA and math was not significant after accounting for the effects of the mediators and confounders (p = 0.796). Whereas in the language model, fitness explained 32% of the association, also in this model the association between VPA and language grade was no longer significant after taking the mediators and confounders into account (p = 0.215). Estimates from the crude version of the structural model are provided in the supporting information (S7 Table).

### 3.6. Sensitivity analyses

Given the differences in the outcomes between those with and without valid accelerometer measurements, sensitivity analyses were conducted, incorporating all participants with at least one valid day. The overall results aligned with those obtained from the models using the more stringent criterion of three valid days. Additionally, BMI was tested as a covariate in the models, but since its inclusion did not significantly affect the results, it was not included in the final model.

Sensitivity analyses were also conducted, excluding adolescents with a late start of the cognitive test battery, as this group had lower scores. The findings were consistent with those obtained when including all participants. Lastly, sensitivity analyses were run including class noise level as a confounder, these results were also in line with those excluding this confounder.

## 4. Discussion

In this study, we investigated the associations between PA (MPA, VPA), fitness, and cognitive or academic performance in Swedish adolescents. We found a positive association between fitness and both cognitive and academic performance. Furthermore, cognitive performance mediated the association with academic performance. Moreover, fitness served as a mediator in the association between VPA and academic performance, although this analysis was done post hoc and the direct association between VPA and academic performance was not significant.

One unique aspect of our study is that we assessed PA, fitness, cognitive, and academic performance within the same sample of adolescents. When examining the results, fitness stands as the only significant factor, showing a positive association with both cognitive and academic performance (grades in math and language). Given the close link between cognitive and academic performance [5,8], it is reasonable to expect that robust cognitive skills would improve comprehension and problem-solving, ultimately resulting in better school grades. Therefore, we hypothesized cognitive performance to act as a mediator. This hypothesis (H7) was supported, as cognitive performance was found to have a mediating role in the association between fitness and academic performance. This result highlights that fitness might not only be an important marker of physical health but also of cognitive performance, which in turn may have implications for academic performance [37].

The mediating role of cognitive performance in the association between fitness and academic performance has been reported in previous cross-sectional studies involving older adolescents [37], and children, for instance in a study by Van der Niet et al. [38]. Van der Niet et al., suggested that such findings prompt the question of whether sufficient executive functioning is necessary before fitness can effectively enhance academic achievement [38]. However, the observational nature of these studies prevents causal claims. Intervention studies in younger children have shown that the effect of acute PA on cognitive performance varies depending on baseline cognitive performance. Specifically, those with lower cognitive performance demonstrated greater improvement, while no change was observed among those with higher baseline performance [24]. Furthermore, a school PA intervention in children found no overall effects on academic performance, yet subgroup analysis showed a positive intervention effect among those with low numeracy skills at baseline [23]. These findings highlight the need to consider cognitive performance when examining the link between fitness or PA and academic performance.

However, it is also important to acknowledge the complexity of these associations and the potential influence of other factors. According to Cognitive Load Theory, information overload can impair working memory, hindering learning, even for individuals with high cognitive abilities [39]. Effective teaching, however, can mitigate this by for instance connecting new information with prior knowledge stored in long-term memory, helping to avoid overwhelming cognitive resources and improving academic performance [39]. Thus, there is an interplay between cognitive abilities, such as working memory and long-term memory, and teaching strategies that influence academic outcomes. Furthermore, the socio-ecological framework states that individual outcomes, such as academic performance or physical activity habits are shaped by multiple, nested environmental systems [40,41]. In the academic environment, these systems range from microsystems (e.g., peer and teacher interactions) to macrosystems (e.g., educational policies). Our model, focusing solely on student-level characteristics, does not account for these broader influences or the bidirectional relationship between academic and cognitive performance. Therefore, future studies should incorporate variables from multiple ecological levels to provide a more nuanced understanding of the diverse factors influencing academic performance, while also investigating causal inferences through longitudinal or randomized controlled trial designs.

Given the numerous factors influencing cognitive and academic performance, such as age [5], parental and environmental factors [6], genetics [7], and engagement and the quality of education received [4] it is not surprising that the associations between fitness and cognitive or academic performance have been reported to be weak [4], especially after controlling for confounding variables [16]. In our study, the largest standardized beta was found in the association between fitness and academic performance, with a 0.16 increase in standardized language grade or 0.02 on the unstandardized grade variable in response to a one-unit increase in fitness. However, it is important to emphasize that the units of fitness are small (1 mL/kg/min). To illustrate, if a student with low fitness (35 ml/kg/min) were to increase fitness by 15 units, this would correspond to a 0.33 increase in language grade, ranging from 1–6. This stresses caution in attributing significant academic improvement solely to fitness enhancements.

Previous studies often lack detailed measures of PA, especially among adolescents [15]. In our study, we addressed this by utilizing accelerometer-based PA, which enabled analysis of different PA intensities (MPA and VPA). Coe et al. have suggested that MPA might not reach the intensity threshold necessary for improvements in academic performance [26]. Therefore, when MPA and VPA are combined into MVPA, such distinction could get lost. Regardless, in the current study, no significant associations between either MPA or VPA and cognitive or academic performance was found. These results contrast with some previous studies, that reported VPA (but not MPA) to be positively related to academic performance in children [26] and older adolescents, although this was only observed among girls [42]. The discrepancy between our findings and the study in older adolescents could be that the associations were gender specific, given that significant associations were only found among girls [42].

Considering that engaging in regular PA typically leads to improved fitness levels [11], it might be surprising that fitness was found to be a significant exposure (despite the weak association), whereas VPA did not. One possible reason could be that PA was measured for one week, offering a snapshot of habits, while fitness, specifically cardiorespiratory endurance, refers to “the ability of the circulatory and respiratory systems to supply fuel during sustained PA” [43], and is therefore an ability developed through consistent PA over time. Further, grades were obtained at the end of grade 6, two to six months before measuring fitness and PA, reflecting the efforts and abilities demonstrated by adolescents throughout the entirety of grade 6. This discrepancy in timing may partly explain why the findings were more conclusive for fitness.

Furthermore, it is important to acknowledge that VPA and fitness represent different constructs. VPA reflects a behavior, while fitness represents a physical ability, also influenced by genetics, especially before puberty [44]. It is plausible that children and adolescents with a genetic disposition towards high fitness, also share a predisposition to specific structural brain features that contribute to enhanced cognitive performance. For example, studies have found that children with high fitness levels had larger volumes of brain structures (such as the basal ganglia [45] and hippocampus [46] associated with higher cognitive performance [45,46]. Furthermore, it has been suggested that high-fit children [47] and adolescents [14] are able to perform better on cognitive tasks through the allocation of more attentional resources, compared to their less-fit counterparts [14,47].

Considering the link between VPA and fitness, it could be inferred that an individual’s fitness level would partially explain the association between VPA and academic performance. The exploratory hypothesis (H8) in this study, that fitness mediates the association between VPA and academic performance, was supported. Although this hypothesis was not pre-registered and therefore represents post hoc theorizing, it was informed by evidence from previous studies in children and adolescents [12,13]. The results showed that in the associations between VPA and academic performance, the mediation models showed that fitness accounted for 77% in math and 32% in language. These findings are in line with previous studies among children [48] and adolescents, although this was only found in boys, [49]. However, these studies used self-reported PA which limits the possibility of distinguishing between PA intensities. The results of the current study indicate that adolescents engaging in more VPA exhibit higher fitness levels and achieve higher grades. However, the direct association between VPA and academic performance was not significant, suggesting that VPA may only be important if considered in the context of fitness. While the cross-sectional design of this study prevents us from determining causal relationships, previous longitudinal studies have shown that students who maintain or enhance their fitness over time tend to exhibit higher academic performance compared to those with lower fitness levels [15]. Although there has been discussion regarding the potential to improve fitness before puberty, a meta-review concluded that PA interventions improved cardiorespiratory fitness among adolescents [11].

Our findings showed that fitness was significantly associated with both cognitive and academic performance, although the associations were weak. Additionally, it was observed that the associations between VPA and academic performance operated through fitness. Therefore, it is concerning that we also found that adolescents having parents with short education, or parents born abroad had lower levels of fitness, PA, cognitive, and academic performance, compared to those having parents with long education or born in Sweden. Therefore, future PA interventions should prioritize the inclusion of these groups. Moreover, to investigate whether enhancing PA and fitness levels among these groups could also improve their academic performance.

A strength of the current study is the relatively large sample, using robust estimates of PA (accelerometry), fitness, and cognitive performance (a comprehensive test battery). Another strength is that we investigated the associations between PA, fitness, cognitive, and academic performance in the same sample, which facilitated direct comparisons across various outcomes. While this is a strength, it is also a limitation as the increased number of models raises the risk of Type I errors. Therefore, we adjusted the p-values for multiple comparisons, however this may increase the risk of Type II errors. Concerning generalizability, this study included adolescents with an average age of 13 years, limiting the possibility to extend these findings to other age groups due to the rapid mental and physical development occurring during this period. A limitation of our study is the potential for selection bias, as participants may not fully represent the broader adolescent population. However, by recruiting adolescents through schools and employing an inclusive strategy, such as translating materials and creating a recruitment film, we aimed to reduce this bias, achieving a diverse sample with a 73% response rate. Regarding measurement bias, we used accelerometers to measure PA, a method considered more reliable than self-reports, as adolescents tend to overestimate their PA [20]. However, accelerometers depend on wear-time compliance, in this study, we required at least three valid days of wear, including one weekend day, as commonly used in previous research [29]. While necessary, this criterion reduced the sample size by 200–300 participants, which may have increased the risk of selection bias. This reduction in sample size likely diminished the statistical power of the study, potentially affecting the reliability of the results. However, it is important to emphasize that this criterion may have led to the non-random exclusion of less compliant or less active adolescents, potentially introduced bias to the results. To address this concern, we conducted sensitivity analyses including all participants with at least one valid day of accelerometer data. These analyses showed results consistent with the primary models. Another consideration is that the use of percentage time spent in PA, may not fully capture meaningful differences in absolute PA levels given the variability in accelerometer wear time across participants, and this should be considered when interpreting the results. Finally, it is essential to acknowledge that this study has a cross-sectional design. Although mediation analyses were conducted within a theoretical framework, causality cannot be inferred, as temporal ordering among fitness, cognition, and academic performance cannot be established. Nonetheless, similar cross-sectional studies in younger populations have used mediation approaches to explore potential pathways [12], providing valuable preliminary insights. Our analyses should therefore be interpreted as exploratory and hypothesis-generating. Longitudinal and experimental studies are needed to clarify temporal and causal relationships among these variables.

## 5. Conclusion

In summary, our results showed that adolescents with higher fitness levels also had higher cognitive and academic performance (grades in math and language). Moreover, cognitive performance had a mediating role in this association, while fitness acted as a mediator in the association between VPA and academic performance. The results also showed lower fitness, PA, cognitive, and academic performance levels among adolescents having parents with short education or born abroad. Therefore, these groups should be considered when planning future interventions aiming at improving academic performance.

## Supporting information

S1 Fig Overview of recruitment of participating schools and adolescents. (TIF)

S2 Fig Overview of the working memory task. (TIF)

S3 Fig Visual overview of the numerical 3-back task. (TIF)

S4 Fig Visual overview of the spatial updating task. (TIF)

S5 Fig Visual overview of object-position recall task. (TIF)

S6 Fig Visual representation of the SEM measurement model of memory using standardized estimates. (TIF)

S1 Table Descriptive characteristics of cognitive test parameters for the whole sample and stratified by gender (n = 1139). (DOCX)

S2 Table Descriptive characteristics of the study sample by parental education group (mean ± SD unless otherwise specified). (DOCX)

S3 Table Descriptive characteristics of the study sample, by parental country of birth (mean ± SD unless otherwise specified). (DOCX)

S4 Table Associations between predictors and memory (episodic memory and working memory) analyzed with multi-level linear regression treating schools as a cluster. (DOCX)

S5 Table Associations between predictors and overall academic performance (grades in language and math) analyzed with multi-level linear regression treating schools as a cluster. (DOCX)

S6 Table Associations between MVPA and cognitive performance (working memory and episodic memory) and academic performance (grade in math and language) analyzed with multi-level linear regression treating schools as a cluster. (DOCX)

S7 Table Crude structural model showing direct and indirect associations between VPA, fitness, cognitive performance, and academic performance (grades in math, n = 729 and language n = 755) using fitness and cognitive performance as mediators. (DOCX)

## Abbreviations

PA: Physical activity
MVPA: Moderate to vigorous physical activity
MPA: Moderate physical activity
VPA: Vigorous physical activity
SEM: Structural equation modeling
RMSEA: Root Mean Square Error of Approximation
CFI: Comparative Fit Index
