# Supplementary material for: Associations between physical activity, fitness, cognitive and academic performance in Swedish adolescents: Findings from a cross-sectional study
Source: PLoS One. 2026 Mar 9;21(3):e0344087. doi: 10.1371/journal.pone.0344087 (PMC12970885; doi:10.1371/journal.pone.0344087)
Supplement: S1 Table — (DOCX) [file pone.0344087.s007.docx]

| **S1 Table.** Descriptive characteristics of cognitive test parameters for the whole sample and stratified by gender (*n*=1139) | | | | | | | | |
| --- | --- | --- | --- | --- | --- | --- | --- | --- |
|  | **n** | **missing** | **All** | **n** | **Girls** | **n** | **Boys** | **Sig.** |
| **Cognitive test battery start time** | 1139 | 0 |  | 580 |  | 558 |  | 0.689 |
| 8-9 AM |  |  | 460 (40.4) |  | 242 (41.7) |  | 217 (38.9) |  |
| 9-10 AM |  |  | 127 (11.2) |  | 65 (11.2) |  | 62 (11.1) |  |
| 10-11 AM |  |  | 367 (32.2) |  | 178 (30.7) |  | 189 (33.9) |  |
| 11-12 AM |  |  | 185 (16.2) |  | 95 (16.4) |  | 90 (16.1) |  |
| **Time duration cog test** | 1138 | 1 |  | 579 |  | 558 |  |  |
| Hours (hh.mm) |  |  | 1.44 (0.07) |  | 1.45 (0.07) |  | 1.44 (0.08) | 0.710 |
| **Classroom noise level during cognitive task** ^3^ | 1117 | 22 |  | 567 |  | 549 |  | 0.339 |
| Low |  |  | 603 (54.0) |  | 318 (56.1) |  | 284 (51.7) |  |
| Medium |  |  | 351 (31.4) |  | 171 (30.2) |  | 180 (32.8) |  |
| High |  |  | 163 (14.6) |  | 78 (13.8) |  | 85 (15.5) |  |
| The descriptives are mean ± standard deviations, unless otherwise specified.  Group differences between boys and girls were analyzed with a t-test (continuous variables) or Chi^2^ test (categorical variables)  ^2^ Perceived classroom noise level | | | | | | | | |
