# Supplementary material for: Associations between physical activity, fitness, cognitive and academic performance in Swedish adolescents: Findings from a cross-sectional study
Source: PLoS One. 2026 Mar 9;21(3):e0344087. doi: 10.1371/journal.pone.0344087 (PMC12970885; doi:10.1371/journal.pone.0344087)
Supplement: S2 Table — (DOCX) [file pone.0344087.s008.docx]

| **S2 Table.** Descriptive characteristics of the study sample by parental education group (mean ± SD unless otherwise specified) | | | | | |
| --- | --- | --- | --- | --- | --- |
|  |  | **Parental education** | | | |
|  | n | ≤ 12 years | n | > 12 years | Sig. |
| **MPA** |  |  |  |  |  |
| MPA (average min per day/week) | 291 | 31.5 (10.2) | 586 | 30.0 (9.6) | **0.033** |
| % MPA (average percentage per day/week) | 291 | 4.0 (1.3) | 586 | 3.8 (1.2) | **0.016** |
|  |  |  |  |  |  |
| **VPA** |  |  |  |  |  |
| VPA (average min per day/week) | 291 | 20.6 (12.2) | 586 | 21.6 (11.8) | 0.253 |
| %VPA (average percentage per day/week) | 290 | 2.6 (1.5) | 586 | 2.7 (1.5) | 0.197 |
|  |  |  |  |  |  |
| **Accelerometer wear time** |  |  |  |  |  |
| Wear time (average week) | 291 | 789.3 (61.7) | 586 | 794.6 (59.9) | 0.230 |
| Total included valid days | 291 | 5.9 (1.1) | 586 | 6.1 (1.0) | **0.037** |
|  |  |  |  |  |  |
| **Fitness** |  |  |  |  |  |
| Estimated V0_2_ max (mL/kg/min) | 331 | 47.4 (10.0) | 657 | 50.6 (9.9) | **<0.001** |
|  |  |  |  |  |  |
| **Working memory score** |  |  |  |  |  |
| Letter updating | 371 | 33.5 (8.1) | 728 | 36.7 (7.0) | **<0.001** |
| Numerical nback | 372 | 67.4 (28.8) | 728 | 79.1 (24.4) | **<0.001** |
| Spacial updating | 372 | 11.0 (6.9) | 728 | 12.8 (6.2) | **<0.001** |
|  |  |  |  |  |  |
| **Episodic memory score** |  |  |  |  |  |
| Word recall | 356 | 14.0 (5.4) | 716 | 16.9 (5.9) | **<0.001** |
| Number-word recall | 365 | 2.6 (2.4) | 724 | 3.6 (2.9) | **<0.001** |
| Object-position recall | 364 | 12.1 (5.2) | 716 | 13.6 (5.2) | **<0.001** |
|  |  |  |  |  |  |
| **Math grade (final grade)** | 359 |  | 704 |  | **<0.001** |
| A, n (%) |  | 19 (5.3) |  | 121 (17.2) |  |
| B, n (%) |  | 36 (10.0) |  | 165 (23.4) |  |
| C, n (%) |  | 53 (14.8) |  | 158 (22.4) |  |
| D, n (%) |  | 88 (24.5) |  | 133 (18.9) |  |
| E, n (%) |  | 110 (30.6) |  | 104 (14.8) |  |
| F, n (%) |  | 53 (14.8) |  | 23 (3.3) |  |
|  |  |  |  |  |  |
| **Swedish grade (final grade)** | 357 |  | 704 |  | **<0.001** |
| A, n (%) |  | 9 (2.5) |  | 90 (12.8) |  |
| B, n (%) |  | 35 (9.8) |  | 189 (26.9) |  |
| C, n (%) |  | 86 (24.1) |  | 181 (25.7) |  |
| D, n (%) |  | 95 (26.6) |  | 140 (19.9) |  |
| E, n (%) |  | 86 (24.1) |  | 80 (11.4) |  |
| F, n (%) |  | 46 (12.9) |  | 24 (3.4) |  |
| %MPA percent spent in moderate physical activity, percent spent in VPA vigorous physical activity, Fitness estimated vo_2_ max in mL/kg/min  Group differences analyzed with a t-test (continuous variables) or Chi^2^ test (categorical variables) | | | | | |
