# Supplementary material for: Associations between physical activity, fitness, cognitive and academic performance in Swedish adolescents: Findings from a cross-sectional study
Source: PLoS One. 2026 Mar 9;21(3):e0344087. doi: 10.1371/journal.pone.0344087 (PMC12970885; doi:10.1371/journal.pone.0344087)
Supplement: S3 Table — (DOCX) [file pone.0344087.s009.docx]

| **S3 Table.** Descriptive characteristics of the study sample, by parental country of birth (mean ± SD unless otherwise specified) | | | | | | | |
| --- | --- | --- | --- | --- | --- | --- | --- |
|  | **Parental country of birth** | | | | | | |
|  | n | Both parents born in Sweden | n | One parent born in Sweden | n | Both parents born abroad | Sig.  p |
| **MPA** |  |  |  |  |  |  |  |
| MPA (average min per day/week) | 549 | 30.5 (9.6) | 127 | 29.9 (9.7) | 204 | 31.2 (10.2) | 0.461 |
| % MPA (average percentage per day/week) | 549 | 3.8 (1.2) | 127 | 3.8 (1.2) | 204 | 4.0 (1.3) | 0.366 |
|  |  |  |  |  |  |  |  |
| **VPA** |  |  |  |  |  |  |  |
| VPA (average min per day/week) | 549 | 21.7 (12.0) | 127 | 21.5 (12.4) | 204 | 20.5 (11.4) | 0.488 |
| %VPA (average percentage per day/week) | 548 | 2.7 (1.5) | 127 | 2.7 (1.5) | 204 | 2.6 (1.5) | 0.694 |
|  |  |  |  |  |  |  |  |
| **Accelerometer wear time** |  |  |  |  |  |  |  |
| Wear time (average week) | 549 | 795.7 (60.0) | 127 | 786.2 (62.1) | 204 | 788.6 (62.8) | 0.161 |
| Total included valid days | 549 | 6.1 (1.0) | 127 | 6.0 (1.1) | 204 | 5.9 (1.2) | **0.037** |
|  |  |  |  |  |  |  |  |
| **Fitness** |  |  |  |  |  |  |  |
| Estimated V0_2_ max (mL/kg/min) | 503 | 50.2 (9.7) | 121 | 49.2 (9.5) | 184 | 47.4 (10.4) | **0.004** |
|  |  |  |  |  |  |  |  |
| **Working memory score** |  |  |  |  |  |  |  |
| Letter updating (min: 4 max: 48) | 655 | 36.1 (7.1) | 168 | 35.9 (7.4) | 280 | 34.5 (8.4) | **0.011** |
| Numerical nback (min: 0 max: 108) | 656 | 78.0 (24.8) | 168 | 76.0 (25.6) | 280 | 67.0 (30.2) | **<0.001** |
| Spacial updating (min: 0 max: 30) | 656 | 12.5 (6.4) | 168 | 11.9 (5.8) | 280 | 11.6 (7.0) | 0.100 |
|  |  |  |  |  |  |  |  |
| **Episodic memory score** |  |  |  |  |  |  |  |
| Word recall (min: 2 max: 32) | 643 | 16.5 (5.9) | 164 | 16.1 (5.5) | 269 | 14.4 (5.7) | **<0.001** |
| Number-word recall (min: 0 max: 19) | 653 | 3.48 (2.8) | 166 | 3.5 (3.0) | 276 | 2.5 (2.4) | **<0.001** |
| Object-position recall (min: 0 max: 24) | 649 | 13.6 (4.9) | 165 | 13.5 (5.3) | 271 | 11.6 (5.6) | **<0.001** |
|  |  |  |  |  |  |  |  |
| **Math grade (final grade)** | 625 |  | 157 |  | 256 |  |  |
| A, n (%) |  | 94 (15.0) |  | 24 (15.3) |  | 20 (7.8) | **<0.001** |
| B, n (%) |  | 143 (22.9) |  | 29 (18.5) |  | 29 (11.3) |  |
| C, n (%) |  | 135 (21.6) |  | 28 (17.8) |  | 44 (17.2) |  |
| D, n (%) |  | 119 (19.0) |  | 39 (24.8) |  | 57 (22.3) |  |
| E, n (%) |  | 104 (16.6) |  | 29 (18.5) |  | 73 (28.5) |  |
| F, n (%) |  | 30 (4.8) |  | 8 (5.1) |  | 33 (12.9) |  |
|  |  |  |  |  |  |  |  |
| **Swedish grade (final grade)** | 624 |  | 157 |  | 255 |  | **<0.001** |
| A, n (%) |  | 72 (11.5) |  | 15 (9.6) |  | 10 (3.9) |  |
| B, n (%) |  | 149 (23.9) |  | 31 (19.8) |  | 42 (16.5) |  |
| C, n (%) |  | 170 (27.2) |  | 45 (28.7) |  | 46 (18.0) |  |
| D, n (%) |  | 136 (21.8) |  | 33 (21.0) |  | 60 (23.5) |  |
| E, n (%) |  | 77 (12.3) |  | 27 (17.2) |  | 58 (22.8) |  |
| F, n (%) |  | 20 (3.2) |  | 6 (3.8) |  | 39 (15.3) |  |
| %MPA percent spent in moderate physical activity, percent spent in VPA vigorous physical activity, Fitness estimated vo_2_ max in mL/kg/min  Group differences analyzed with a one-way ANOVA (continuous variables) or Chi^2^ test (categorical variables) | | | | | | | |
