# Supplementary material for: Associations between physical activity, fitness, cognitive and academic performance in Swedish adolescents: Findings from a cross-sectional study
Source: PLoS One. 2026 Mar 9;21(3):e0344087. doi: 10.1371/journal.pone.0344087 (PMC12970885; doi:10.1371/journal.pone.0344087)
Supplement: S4 Table — (DOCX) [file pone.0344087.s010.docx]

| **S4 Table.** Associations between predictors and memory (episodic memory and working memory) analyzed with multi-level linear regression treating schools as a cluster | | | | | | | | |
| --- | --- | --- | --- | --- | --- | --- | --- | --- |
| **Model** | **Cognitive performance** | | | | | | | |
|  | Working memory | | | | Episodic memory | | | |
| **Crude** | n | b (95% CI) | Sig  (p) | β | n | b (95% CI) | Sig  (p) | β |
| %MPA | 864 | -0.15 (-0.31, 0.00) | 0.051 | -0.84 | 864 | **-0.10 (-0.16, -0.03)** | **0.005** | **-0.09** |
| %VPA | 863 | 0.01 (-0.11, 0.13) | 0.729 | 0.01 | 863 | 0.00 (-0.06, 0.07) | 0.893 | 0.01 |
| Fitness | 980 | 0.00 (-0.01, 0.02) | 0.780 | 0.01 | 980 | -0.00 (-0.01, 0.01) | 0.658 | -0.01 |
| **Adjusted** |  |  |  |  |  |  |  |  |
| %MPA | 766 | -0.051 (-0.20, 0.09) | 0.493 | -0.03 | 766 | -0.05 (-0.12, 0.01) | 0.117 | -0.05 |
| %VPA | 765 | 0.07 (-0.04, 0.18) | 0.197 | 0.04 | 765 | 0.04 (-0.03, 0.10) | 0.251 | 0.04 |
| Fitness | 865 | **0.03 (0.01, 0.05)** | **0.008** | **0.11** | 865 | **0.01 (0.00, 0.02)** | **0.010** | **0.11** |
| The adjusted model included parental education, parental country of birth, pubertal status and gender as confounders  Coefficients: b= unstandardized and β= standardized; CI; confidence interval,  Abbreviations: Fitness; Estimated V02 max expressed in mL/kg/min, %MPA; percent spent in moderate physical activity, %VPA; percent spent in VPA vigorous physical activity.  Working memory and Episodic memory are based on factor scores from a SEM model loading from 2 latent factors: episodic memory and working memory, with 3 tests in each domain. | | | | | | | | |
