# Supplementary material for: Associations between physical activity, fitness, cognitive and academic performance in Swedish adolescents: Findings from a cross-sectional study
Source: PLoS One. 2026 Mar 9;21(3):e0344087. doi: 10.1371/journal.pone.0344087 (PMC12970885; doi:10.1371/journal.pone.0344087)
Supplement: S5 Table — (DOCX) [file pone.0344087.s011.docx]

| **S5 Table.** Associations between predictors and overall academic performance (grades in language and math) analyzed with multi-level linear regression treating schools as a cluster | | | | |
| --- | --- | --- | --- | --- |
| **Overall academic performance** | | | | |
| **Crude** | n | b (95% CI) | Sig  (p) | β |
| %MPA | 852 | **-0.64 (-1.12, -0.16)** | **0.009** | **-0.09** |
| %VPA | 851 | 0.29 (-0.25, 0.83) | 0.293 | 0.05 |
| Fitness | 914 | 0.06 (0.00, 0.11) | 0.036 | 0.06 |
| **Adjusted** |  |  |  |  |
| %MPA | 772 | -0.35 (-0.78, 0.08) | 0.112 | -0.05 |
| %VPA | 771 | 0.45 (-0.06, 0.97) | 0.084 | 0.07 |
| Fitness | 867 | **0.17 (0.08, 0.25)** | **<0.001** | **0.18** |
| Coefficients: b= unstandardized and β= standardized; CI; confidence interval,  Abbreviations: Fitness; Estimated V0_2_ max expressed in mL/kg/min, %MPA; percent spent in moderate physical activity, %VPA; percent spent in VPA vigorous physical activity.  Overall academic performance was based on the average T-score from the final grade for language (Swedish) and Math in grade 6.  The adjusted model included parental education, parental country of birth, pubertal status, and gender as confounders | | | | |
