# Supplementary material for: Associations between physical activity, fitness, cognitive and academic performance in Swedish adolescents: Findings from a cross-sectional study
Source: PLoS One. 2026 Mar 9;21(3):e0344087. doi: 10.1371/journal.pone.0344087 (PMC12970885; doi:10.1371/journal.pone.0344087)
Supplement: S6 Table — (DOCX) [file pone.0344087.s012.docx]

| **S6 Table.** Associations between MVPA and cognitive performance (working memory and episodic memory) and academic performance (grade in math and language) analyzed with multi-level linear regression treating schools as a cluster | | | | | | | | | | |
| --- | --- | --- | --- | --- | --- | --- | --- | --- | --- | --- |
| **Cognitive performance** | | | | | | | | | | |
| %MVPA | n | | b (95% CI) | | | sig (p) | | β | | |
| Crude | 818 | | 0.03 (-0.27, 0.34) | | | 0.833 | | 0.01 | | |
| Adjusted^1^ | 798 | | 0.00 (-0.02, 0.03) | | | 0.851 | | 0.01 | | |
| **Academic performance** | | | | | | | | | | |
|  | Maths | | | | | Language (Swedish) | | | | |
| %MVPA | n | b (95% CI) | | sig (p) | β | n | b (95% CI) | | sig (p) | β |
| Crude | 854 | 0.03 (-0.27, 0.33) | | 0.845 | 0.01 | 852 | -0.01 (-0.06, 0.03) | | 0.522 | -0.03 |
| Adjusted^1^ | 742 | 0.05 (-0.26, 0.36) | | 0.758 | 0.01 | 772 | 0.02 (-0.02, 0.06) | | 0.271 | 0.04 |
| Coefficients: b= unstandardized and β= standardized; CI; confidence interval,  ^1^The adjusted model included parental education, parental country of birth, pubertal status, and gender as confounders  The cognitive performance outcome are based on factor scores from a SEM model loading from 2 latent factors: episodic memory and working memory, with 3 tests in each domain.  Abbreviations: Fitness; Estimated V0_2_ max expressed in mL/kg/min, %MPA; percent spent in moderate physical activity, %VPA; percent spent in VPA vigorous physical activity. | | | | | | | | | | |
