# Supplementary material for: Associations between physical activity, fitness, cognitive and academic performance in Swedish adolescents: Findings from a cross-sectional study
Source: PLoS One. 2026 Mar 9;21(3):e0344087. doi: 10.1371/journal.pone.0344087 (PMC12970885; doi:10.1371/journal.pone.0344087)
Supplement: S7 Table — (DOCX) [file pone.0344087.s013.docx]

| **S7 Table.** Crude structural model showing direct and indirect associations between VPA, fitness, cognitive performance, and academic performance (grades in math, n=729 and language n=755) using fitness and cognitive performance as mediators | | | | | | | | | | | |
| --- | --- | --- | --- | --- | --- | --- | --- | --- | --- | --- | --- |
|  | | | | | | | | | | | |
|  |  | Direct association | | |  | Indirect associations through the mediator (fitness) | | | | | |
| Predictor | Outcome | β | SE | 95% CI | Sig  p | β | SE | 95% CI | Sig  p | RIT | RID |
| %VPA | Math grade | 0.038 | 0.03 | -0.020, 0.096 | 0.203 | 0.039 | 0.010 | 0.021, 0.059 | <0.001 | 0.506 | 1.023 |
|  | Language grade | 0.055 | 0.04 | -.018, 0.127 | 0.139 | -0.002 | 0.009 | -0.021, 0.017 | 0.809 | 0.048 | 0.045 |
|  |  |  |  |  |  |  |  |  |  |  |  |
| Predictor | Outcome | Direct association | | |  | Indirect associations through the mediator (cognitive performance) | | | | | |
|  |  | β | SE | 95% CI | Sig  p | β | SE | 95% CI | Sig  p | RIT | RID |
| Fitness | Math grade | 0.135 | 0.03 | 0.079, 0.192 | <0.001 | 0.049 | 0.018 | 0.014, 0.086 | 0.007 | 0.266 | 0.362 |
|  | Language grade | -0.009 | 0.03 | -0.072, 0.055 | 0.791 | 0.046 | 0.017 | 0.013, 0.080 | 0.007 | 1.232 | 5.313 |
| β, standardized beta coefficient; SE, standard error; RIT, ratio of the indirect effect to the total effect; RID, ratio of the indirect effect to the direct effect; %VPA, percent spent in VPA vigorous physical activity; Fitness, Estimated V02 max expressed in mL/kg/min. | | | | | | | | | | | |
